# Molecular docking analysis of N-myristoyl-transferase with small molecules from the ZINC database for screening potential anti-malarial drugs

**DOI:** 10.6026/973206300211397

**Published:** 2025-06-30

**Authors:** Anbuselvam Mohan, Katherine C. Ji, Balasubramanian Chitra, Anbuselvam Jeeva, Hai-Feng Ji

**Affiliations:** 1 Department of Biotechnology, SrimadAndavan Arts and Science, Tiruchirappalli, Tamil Nadu, India; 2 Department of Chemistry, Drexel University, Philadelphia, PA 19104, USA; 3 Department of Microbiology, Imayam Arts and Science College, Tiruchirappalli, Tamilnadu, India

**Keywords:** Malaria, *Plasmodium vivax*, N-Myristoyltransferase (NMT), virtual screening, molecular dynamics simulation

## Abstract

Malaria is one of the major global public health problems, is primarily caused by protozoan parasites of the genus *Plasmodium* and transmitted through the bites of infected female *Anopheles* mosquitoes. N-Myristoyltransferase (NMT) is an important drug target, particularly for *Plasmodium vivax*. Therefore, it is of interest to describe the molecular docking analysis of N-myristoyl-transferase with small molecules from the ZINC database for screening potential anti-malarial drugs. Hence, we report four potential compounds namely ZINC37555319, ZINC41016284, ZINC41016205, and ZINC47160805 with acceptable ADME properties for further consideration.

## Background:

Protozoan parasites are significant causative agents of infectious diseases globally, with malaria being one of the most prevalent and life-threatening. Annually, millions are affected, particularly in tropical and subtropical regions, with children under five and pregnant women being the most vulnerable. According to the WHO, an estimated 229 million malaria cases and 409,000 related deaths were reported in 2020 [1], with children under five accounting for 67% of fatalities. Malaria is caused by *Plasmodium* species (*P. falciparum*, *P. vivax*, *P. ovale*, *P. malariae*, and *P. knowlesi*), transmitted through the bites of female *Anopheles* mosquitoes. Following transmission, the malaria parasites travel through the bloodstream to the liver, where they infect hepatocytes. During the intracellular stage of the infection, the parasite replicates its genome over 2-10 days, producing tens of thousands of daughter parasites known as merozoites. Once mature, these parasites return to the bloodstream, where they cause symptomatic malaria. Efforts to control and prevent malaria have led to a 37% reduction in annual global malaria infections and a 60% decrease in mortality rates. However, since 2014, malaria cases have steadily risen in Africa due to the emergence of drug-resistant parasitic strains [2]. Among these, *P. falciparum* and *P. vivax* are the most pathogenic, with *P. vivax* being the leading cause of malaria-related deaths in Sub-Saharan Africa [3]. Common symptoms include fever, chills, fatigue, headache, a bitter taste in the mouth, vomiting, diarrhea, convulsions, anemia, hemoglobinuria ("Coca-Cola urine"), hypoglycemia, prostration, and hyperpyrexia [4, 5]. Malaria research has extensively focused on different stages of the parasite's life cycle, as targeting regulatory proteins involved in parasite transmission could provide robust strategies for disease eradication. One such protein, N-Myristoyltransferase (NMT), is present in eukaryotes and was first discovered in Saccharomyces cerevisiae. NMT is a monomeric enzyme responsible for N-myristoylation, a process involving the co- and post-translational attachment of the C14 fatty acid myristic acid to the N-terminal glycine of a substrate protein via an amide bond. This process plays a critical role in forming the inner membrane complex, which is essential for the parasite's life cycle. Several proteins are involved in the N-myristoylation process. In malaria, proteins such as calcium-dependent protein kinase 14 and glideosome-associated protein 455 rely on myristoylation to perform their biological functions. Inhibiting NMT activity disrupts the N-myristoylation pathway, causing pleiotropic effects that ultimately lead to rapid and irreversible death of the parasite. NMT is, therefore, a well-validated drug target for the development of antimalarial therapies. Several research groups have explored potential NMT inhibitors using computational approaches [6, 7-8] and some previously identified inhibitors have shown activity against the blood stage of *P. vivax*. However, *P. vivax* has developed resistance to several previously effective drugs, including chloroquine, primaquine and sulphadoxine-pyrimethamine [9]. This has prompted an urgent need for new antimalarial drugs to address resistance and achieve effective malaria eradication [10]. Therefore, it is of interest to desciebe the molecular docking analysis of N-myristoyl-transferase with small molecules from the ZINC database for screening potential anti-malarial drugs.

## Materials and Methods:

## Virtual screening:

Structure-based virtual screening is widely utilized during the early stages of drug discovery to identify novel and effective ligands with high affinity for therapeutic targets. In this study, the X-ray crystal structure of NMT (PDB ID: 4A95) with a resolution of 1.55 Å was obtained from the Protein Data Bank [11]. The target structure was prepared and refined using the Protein Preparation Wizard implemented in the Schrödinger suite. This preprocessing step involved several criteria: (i) addition of hydrogen atoms, (ii) reconstruction of missing atoms and side chain residues, along with assignment of proper partial charges, (iii) creation of disulfide bonds, (iv) removal of unwanted water molecules from the target structure. Subsequently, the preprocessed protein structure was optimized using PROPKA, and energy minimization was performed with the OPLS3 force field until the root-mean-square deviation (RMSD) of heavy atoms converged to 0.3 Å. The optimized structure was then used in the Glide receptor grid generation panel to define a three-dimensional (3D) grid box with dimensions of 20x20x20 Å. Van der Waals scaling factors were set to 1.0, and the partial charge cut-off was adjusted to 0.25 [12, 13]. For the virtual screening process, approximately 300,000 compounds were downloaded from the ZINC database in SDF format [14]. These structures were imported into the LigPrep panel for preparation, following stringent criteria: (i) ionization points were calculated at pH 7.0 ± 2.0 and stereoisomers were generated for each ligand, (ii) structural energy minimization was performed using the OPLS3 force field, (iii) desalt options were applied, (iv) tautomers were generated for all conformers, and (v) a single lowest-energy conformer was retained per ligand [15]. The prepared protein and ligand structures were then used as input for structure-based virtual screening. Glide, a highly efficient computational tool, was employed to perform the screening through a three-step filtering process: High-Throughput Virtual Screening (HTVS), Standard Precision (SP), and Extra Precision (XP). HTVS efficiently filtered millions of compounds, SP provided reliable docking of tens of thousands of ligands, and XP further refined the selection to eliminate unwanted compounds [16].

## ADME prediction:

Currently, drug discovery research groups invest substantial time and resources into developing effective drug candidates with desired pharmacological activities. However, many chemical agents fail to reach the market or achieve their anticipated pharmacological efficacy against validated therapeutic targets in clinical trials. Nearly 60% of drug candidates fail during clinical trials due to inadequate solubility, permeability, or unacceptable toxicity levels. Molecules with poor ADME (absorption, distribution, metabolism, and excretion) properties often prolong the experimental process. An ideal drug candidate must not only exhibit potent pharmacological activity but also possess critical qualities such as bioavailability and a favorable toxicological profile [17]. Experimental determination of ADME properties for newly developed chemical entities is often impractical due to high costs, lengthy processes, and ethical concerns related to animal testing. Consequently, pharmaceutical researchers increasingly rely on in silico ADME prediction tools to evaluate the drug-likeness of chemical compounds. These tools are widely used in the early stages of drug discovery to identify compounds with favorable ADME profiles, ensuring they can effectively reach the target site, perform their intended action, and avoid accumulation or toxic effects. In this study, we employed QikProp, a widely recommended in silico ADME prediction tool known for its ease of use and reliable output. The following key properties were assessed: partition coefficient (QPlogP, octanol/water), water solubility (QPlogS), gut-blood barrier permeability (QPPCaco2), predicted IC50 for potassium channel blockage (QPlogHERG), human oral absorption percentage, molecular weight, hydrogen bond donors/acceptors, number of rotatable bonds, and Madin-Darby Canine Kidney (MDCK) cell permeability [18]. These parameters provide crucial insights into the drug-likeness and potential success of the evaluated compounds.

## Molecular dynamics simulation:

Biological macromolecules undergo significant conformational changes to activate their essential biological functions. These conformational alterations occur within the organism due to factors such as enzymatic activation, mutations, phosphorylation, protonation, or the binding and unbinding of ligands. Understanding macromolecule-ligand interactions at the atomic level is crucial for gaining insights into the molecular behavior of various biological molecules [19]. In this study, molecular dynamics (MD) simulations were performed using the computational tool Desmond to investigate conformational changes, ligand binding, binding stability, and the variability of the top-ranking docked complexes. The System Builder panel was used to generate an orthorhombic simulation box with the SPC water model. The distance between the protein surface and the simulation box was maintained at 10 Å. The system was then neutralized by adding an appropriate number of Na+ and Cl- ions. Following energy minimization, the system underwent a 100 ns MD simulation under the NPT ensemble, maintaining a constant temperature of 300 K and pressure of 1.01325 bars. Energy and trajectory data were recorded at intervals of 1.2 ps and 100 ps, respectively. The simulation was carried out using the OPLS-3e force field [19]. Finally, the Root Mean Square Deviation (RMSD), Root Mean Square Fluctuation (RMSF), and various intermolecular contacts of the top docked complexes were analyzed using the Simulation Interaction Diagram (SID) panel [20].

## Results and Discussion:

The Compound IDs are labelled as such under the ZINC database; Glide score (Kcal/mol), Glide energy (Kcal/mol); No of hydrogen bond interactions; Interacting residues; Distance between the protein residue and the ligand (Å). Molecular weight (<500 Da). Hydrogen bond donors (<5). Hydrogen bond acceptors (<10). Predicted octanol/water partition co-efficient log p (acceptable range: 22.0 to 6.5). Predicted Caco-2 cell permeability in nm/s (acceptable range: 25 is poor and .500 is great). Predicted IC50 value for blockage of HERG K+ channels (concern: below -5). QP log BB for brain/blood (-3.0 to 1.2). Predicted percentage of human oral absorption (25 % is poor).

## Identification of NMT inhibitors:

To identify potential inhibitors against NMT in the effort to combat malaria, we performed a virtual screening approach using the ZINC database. The Glide tool was employed to carry out virtual screening, enabling rapid ligand filtration, as well as accurate evaluation of binding affinity and efficacy. Glide features a multi-step ligand filtration process, including High-Throughput Virtual Screening (HTVS), Standard Precision (SP), and Extra Precision (XP) modes. To evaluate the docking potency of Glide, we first downloaded the crystal structure of NMT (PDB: 4A95) from the Protein Data Bank. The co-crystal inhibitor quinoline was extracted from the active site of the NMT structure, and then re-docked into the same position using the three Glide docking modes. The binding free energies observed for each docking mode were -5.21 kcal/mol, -7.32 kcal/mol, and -8.43 kcal/mol, which were further utilized for database screening. We then superimposed the native crystal structure with the re-docked structure, and the RMSD of the superimposed structures was found to be 0.213 Å. The residues GLY 386, GLY 199, and ASN 365 were involved in various interactions with ligand molecules. These results demonstrate that the Glide docking protocol is highly dynamic and effective for further screening processes. In the first step of the database screening, the prepared compounds were docked into the active site region of NMT. A total of 50,000 compounds were filtered out using the HTVS mode. Then, 16,450 compounds were selected using SP mode, and 1,003 compounds remained after applying the XP mode. The compounds were further evaluated for ADME drug-like properties, which served as an additional filter. Based on the predicted ADME properties, four potential compounds were selected for NMT inhibition. These compounds were: Compound 1: ZINC37555319 - N-{4-[(benzylcarbamoyl)methyl]phenyl}-N-[(4-methylphenyl)methyl]furan-2-carboxamide; Compound 2: ZINC41016284 - 2-(1,3-Dioxo-1,3-dihydro-isoindol-2-yl)-4-methyl-pentanoic acid [4-(4-fluoro-phenyl)-5-methyl-thiazol-2-yl]-amide; Compound 3: ZINC41016205 - 2-(1,3-Dioxo-1,3-dihydro-isoindol-2-yl)-4-methyl-pentanoic acid [4-(4-fluoro-phenyl)-5-methyl-thiazol-2-yl]-amide; Compound 4: ZINC47160805 - 2-(1,3-Dioxo-1,3-dihydro-isoindol-2-yl)-4-methyl-pentanoic acid [4-(4-fluoro-phenyl)-5-methyl-thiazol-2-yl]-amide. The structures of these hit compounds are shown in Figure 1 (see PDF), and the interacting residues are presented in Table 1 (see PDF). The 3D structures of the compounds in the active site of NMT are depicted in Figure 2 (see PDF).

The docking simulations of the four compounds within the binding site of NMT were analyzed and summarized as follows:

## Binding Mode of ZINC37555319:

Compound ZINC37555319 exhibited the lowest Glide score and formed a single hydrogen bond interaction with NMT. The hydrogen atom of SER 319 interacted with the oxygen atom of the compound, with a bond distance of 2.30 Å. Additionally, two π-π stacking interactions were observed between the phenyl ring of ZINC37555319 and the hydrophobic residues PHE 105 and PHE 103, which enhanced the stability of the ZINC37555319-NMT complex. The residues LEU 330, TYR 95, LEU 388, TYR 315, VAL 96, and PHE 226 primarily contributed to hydrophobic interactions. The Glide score and Glide energy for this compound were -10.20 kcal/mol and -81.74 kcal/mol, respectively.

## Binding Mode of ZINC41016284:

Three hydrogen bond interactions were formed between NMT and ZINC41016284. The hydrogen atom of the compound interacted with the backbone oxygen atoms of GLY 199 and GLY 386, with bond distances of 2.07 Å and 2.28 Å, respectively. In addition to these key residues, LEU 410, TYR 211, LEU 388, TYR 334, VAL 96, PHE 226, and VAL 363 were involved in hydrophobic interactions. The Glide score and Glide energy were -10.15 kcal/mol and -88.81 kcal/mol, respectively.

## Binding Mode of ZINC41016205:

ZINC41016205 formed one hydrogen bond interaction with NMT. The backbone hydrogen atom of GLY 199 interacted with an oxygen atom of ZINC41016205 at a bond distance of 2.02 Å. Hydrophobic interactions involved the residues TYR 95, LEU 410, TYR 211, LEU 388, PHE 162, TYR 334, VAL 96, PHE 226, and VAL 363. The Glide score and Glide energy for this compound were -9.80 kcal/mol and -77.10 kcal/mol, respectively, making ZINC41016205 the compound with the fourth least binding free energy with NMT.

## Binding Mode of ZINC47160805:

Three hydrogen bond interactions were observed between ZINC47160805 and NMT. The first occurred with the side chain hydrogen atom of ASN 365, which tightly interacted with the oxygen atom of ZINC47160805, with a bond distance of 2.05 Å. The second bond involved the backbone hydrogen atom of GLY 199, interacting with the oxygen atom of ZINC47160805 at a distance of 2.02 Å. The third interaction was between the hydrogen atom of TYR and the oxygen atom of ZINC47160805, with a bond distance of 2.45 Å. Additionally, two π-π stacking interactions were found between the phenyl ring of ZINC47160805 and the hydrophobic residues PHE 103 and HIS 213, stabilizing the ZINC47160805-NMT complex. Other hydrophobic interactions involved residues such as LEU 410, TYR 211, LEU 388, TYR 334, VAL 96, PHE 226, and VAL 363. The Glide score and Glide energy for this compound were -9.78 kcal/mol and -69.68 kcal/mol, respectively.

## ADME properties:

The drug-like properties of the four newly identified hit molecules were assessed using QikProp to eliminate undesired compounds early in the drug development process. These four molecules satisfied the key criteria of Lipinski's Rule of Five, including: Molecular weights < 500 Da, fewer than 5 hydrogen bond donors, fewer than 10 hydrogen bond acceptors, a predicted octanol/water partition coefficient (QPlogP_o/w) < 5.All these properties fall well within the acceptable range, confirming that the compounds possess favorable drug-like characteristics. Further drug-like behavior assessments were conducted using QikProp. For the four hit molecules, cell permeability (QPPCaco2)-a critical factor in drug metabolism and membrane permeability-ranged from ~126 to ~1864 nm/s (with an acceptable range of 25 nm/s being poor and 500 nm/s being ideal). The predicted IC50 values for the blockage of HERG K+ channels were within the acceptable range of below -5. The predicted Brain/Blood barrier permeability values ranged from ~-2.023 to ~-0.573, which are within the acceptable limits. Additionally, the predicted percentage of human oral absorption for these compounds ranged from 84% to 100%, which is considered optimal (a value below 25% is poor). All pharmacokinetic parameters of the hit molecules were well within the acceptable ranges, making them promising candidates for further drug research in humans. The results of the ADME properties are summarized in Table 2 (see PDF).

## Molecular dynamics simulation:

To assess the stability, conformational changes, and intermolecular interactions of the hit compounds with NMT, we performed molecular dynamics (MD) simulations using Desmond. Notably, the RMSD plot for ZINC47160805 could not be obtained, indicating that the ZINC47160805-NMT complex was unstable. Therefore, stability analyses for only the three remaining complexes were discussed in the following sections.

## MD trajectory analysis of ZINC37555319-NMT:

The RMSD plot of the ZINC37555319-NMT complex is shown in Figure 3a (see PDF), indicating slight conformational fluctuations at 27.90 ns and 98.50 ns, with RMSD values of 1.61 Å and 1.80 Å, respectively. A slight deviation was also observed in the ligand R MSD at 28.80 ns and 92.80 ns, with RMSD values of 1.49 Å and 1.50 Å. Despite these fluctuations, they did not significantly affect the stability of the ZINC37555319-NMT complex. The bar diagram in Figure 3b (see PDF) illustrates hydrogen bond, hydrophobic, and water bridge interactions. The residue ASN 364 (occupancy: 0.624) was primarily involved in hydrogen bonding, while hydrophobic interactions were observed with residues PHE 105 (0.851) and PHE 103 (0.408). Additionally, a hydrogen bond with ASN 365 (maximum occupancy: 53%) and three water bridge interactions involving residues ASP 98, SER 319, and TYR 334 were noted. One pi-cation interaction between HIS 213 and the furan ring of ZINC37555319 also contributed to the binding stability during the simulation (Figure 3c - see PDF).

## MD trajectory analysis of ZINC41016284-NMT:

The RMSD plot for the ZINC41016284-NMT complex is shown in Figure 4a (see PDF), where no fluctuation in the protein RMSD was observed until 85 ns. However, slight conformational fluctuations occurred at 85.80 ns, with an RMSD value of 2.15 Å. For the ligand, the RMSD deviation increased slightly at 1.60 ns, 17.50 ns, 53 ns, and 99.70 ns, with RMSD values of 3.69 Å, 3.96 Å, 4.53 Å, and 5.58 Å, respectively. The bar diagram in Figure 4b (see PDF) shows hydrogen bond and water bridge interactions. Hydrophobic interactions involved residues PHE 103 (0.748) and PHE 105 (0.613), while water bridge interactions were observed with residue ASP 98 (0.499). Additionally, residues ASN 365 and PHE 103 were involved in hydrogen bond and pi-cation interactions. Water bridge interactions, involving residues ASP 98, HIS 213, SER 319, and TYR 334, contributed to the binding stability with maximum occupancies of 39%, 56%, 39%, 42%, 36%, and 49%, respectively (Figure 4c - see PDF).

## MD trajectory analysis of ZINC41016205-NMT:

The RMSD plot for the ZINC41016205-NMT complex is shown in Figure 5a (see PDF), where slight conformational fluctuations occurred at 97.10 ns, with an RMSD value of 1.92 Å. Ligand RMSD deviations increased from 41.30 ns to 99.10 ns, with values of 5.57 Å and 6.04 Å, respectively. The bar diagram in Figure 5b (see PDF) shows hydrogen bond and water bridge interactions. The residue ASP 99 (maximum occupancy: 0.591) was involved in hydrogen bond interactions. Water bridge interactions were observed with residues PHE 103 (0.814) and ASP 385 (0.427). Notably, the residues ASP 99 and ASP 385 contributed significantly to water bridge interactions, with maximum occupancies of 33% and 51%, respectively. These interactions played an important role in enhancing the stability of the protein-ligand complex during the simulation (Figure 5c - see PDF).

## Conclusion:

The global mortality rate from malaria has increased due to the lack of effective treatment options and the rising prevalence of drug-resistant *Plasmodium* strains. Hence, we four potential inhibitors: ZINC37555319, ZINC41016284, ZINC41016205, and ZINC47160805 with appropriate ADME properties and also the molecular dynamics simulation analysis revealed the top three compounds were more stable throughout the simulation period. Thus, we suggest that these three compounds are promising candidates for further development as drug-like molecules.

## Funding statement:

This research received no external funding.

## References

[1] https://www.who.int/news-room/feature-stories/detail/world-malaria-report-2019.

[2] https://www.who.int/.

[3] Gmanyami J.M (2020). Malar J..

[4] Marco A.B (2013). Bioorg Med ChemLett..

[5] Nureni O.A (2019). Data Brief..

[6] Tayo O.O (2014). Org Biomol Chem..

[7] Anja C.S (2018). ACS Infect Dis..

[8] Megan H.W (2014). Nat Chem..

[9] Marcelo U.F (2021). Int J Parasitol Drugs Drug Resist..

[10] Mohamed H.B (2018). Curr Neuropharmacol..

[11] Victor G (2012). Med Chem..

[12] Norah A.A (2022). Molecules..

[13] Velázquez-Libera  J (2019). Front chem..

[14] Irwin J.J (2005). Journal of Chemical Information and Modeling..

[15] Saravana P.P (2020). Food Front..

[16] Anbuselvam M (2022). Mol Divers..

[17] https://www.schrodinger.com/platform/products/qikprop/.

[18] Anbuselvam M (2021). J Biomol Struct Dyn..

[19] Fu C (2013). Environ Toxicol Pharmacol..

[20] Ramkin E.S (2017). J Biomol Struct Dyn..

